# Effect of radiotherapy interruption on nasopharyngeal cancer

**DOI:** 10.3389/fonc.2023.1114652

**Published:** 2023-04-06

**Authors:** Fangrui Zhao, Dashuai Yang, Xiangpan Li

**Affiliations:** ^1^ Department of Oncology, Renmin Hospital of Wuhan University, Wuhan, Hubei, China; ^2^ Department of Hepatobiliary Surgery, Renmin Hospital of Wuhan University, Wuhan, China

**Keywords:** nasopharyngeal carcinoma, radiotherapy, interruption, mechanism, NPC

## Abstract

Nasopharyngeal carcinoma (NPC) is a malignant tumor originating from the epithelial cells of the nasopharynx with a unique geographic distribution, and is particularly prevalent in East and Southeast Asia. Due to its anatomical location, the surgery is difficult to access and the high sensitivity of nasopharyngeal cancer to radiotherapy (RT) makes it the main treatment modality. Radical radiotherapy is the first-line treatment for early-stage nasopharyngeal carcinoma and the cornerstone of multidisciplinary treatment for patients with locally advanced nasopharyngeal carcinoma. Nevertheless, radiotherapy interruption is inevitable as a consequence of unavoidable factors such as public holidays, machine malfunction, patient compliance, and adverse response to treatment, which in turn leads to a reduction in bioactivity and causes sublethal loss of tumor cells to repair. Unirradiated tumor cells are more likely to repopulate at or near their original fastest growth rate during this interval. If no measures are taken after the radiotherapy interruption, such as increasing the dose of radiotherapy and systemic therapy, the tumor is most likely to go uncontrolled and then progress. This review describes the effects of radiotherapy interruption on nasopharyngeal carcinoma, the mechanism of the effect, and explores the measures that can be taken in response to such interruption.

## 1 Introduction

Nasopharyngeal carcinoma (NPC), a malignant tumor, originates from the epithelial cells of the nasopharynx, and is characterized by a unique geographical location with particular prevalence in East and Southeast Asia (1). The incidence can be as high as 25 to 50 cases per 100,000 in southern China (2). According to the International Agency for Research on Cancer (IARC), approximately 129,000 people were diagnosed with nasopharyngeal cancer in 2018, which only accounts for 0.7% of total cancers diagnosed (3). Gender differences exist in the incidence of nasopharyngeal cancer, with a higher incidence in males than in females, and the ratio was approximately 2.5:1 in China in 2015 (4).

The World Health Organization (WHO) classifies nasopharyngeal carcinoma into three histological subtypes, namely keratinizing squamous cell carcinoma, nonkeratinizing (differentiated or undifferentiated) carcinoma, and basal-like carcinoma. Undifferentiated carcinomas are the most common in high prevalence areas, accounting for about 95% or more (1, 5, 6). Nasopharyngeal carcinoma may be associated with Chinese salt cured fish, passive smoking, oral health and oral microbiota as well as with infection of Epstein-Barr virus (EBV) of infection (7).

Compared with computed tomography (CT), magnetic resonance imaging (MRI) can better identify early-stage nasopharyngeal carcinoma, with greater sensitivity and discrimination of infiltration of adjacent soft tissue, skull base and cranial nerve infiltration, and involvement of retropharyngeal lymph nodes. With its advantages of high soft tissue resolution, multiparametric imaging and non-ionizing radiation, MRI has replaced CT as the first choice for diagnosis, staging, efficacy assessment and follow-up of nasopharyngeal carcinoma (8). Surgery is difficult to operate owing to its specific anatomical location. In contrast, nasopharyngeal carcinoma is highly sensitive to radiotherapy (RT), making it the primary treatment modality. Radical radiotherapy is the first-line treatment for early-stage nasopharyngeal carcinoma and the cornerstone of multi-disciplinary treatment for patients with locally advanced nasopharyngeal carcinoma (9, 10).

## 2 Development of radiotherapy

Conventional two-dimensional radiotherapy (2DCRT) was the main radiotherapy technique until the 1990s. 2DCRT is principally based on contraction field radiation techniques, where the target field is gradually reduced or modified to deliver the desired dose (11). However, conventional radiotherapy of the head and neck is associated with severe acute and late toxicity due to the limitations of its degree of consistency. Mucositis is the most common acute side effect caused by radiation to the oral mucosa, accompanied by severe pain, dysphagia and malnutrition. Other acute and late effects include xerostomia and taste disturbances, hearing loss, persistent xerostomia, radiological osteonecrosis of the mandible, and dysphagia (12–14). Although there is no significant impact on survival outcomes, quality of life can be severely diminished.

Over the past decade, intensity-modulated radiotherapy (IMRT) has replaced 2DCRT, which uses a dynamic multileaf collimator to adjust the shape and intensity of individual beams to achieve optimal dose distribution in the tumor region. A more conformal dose distribution allows IMRT to minimize dose delivery to organs at risk (OAR), including the brainstem, spinal cord, and optic cross (15–17). The application of daily image guidance (image-guided radiation therapy) also reduces the dose in the planned target volume (PTV), which further reduces normal tissue exposure (18). Compared to conventional two-dimensional (2D) or three-dimensional (3D) radiotherapy, IMRT provides high doses of radiation for nasopharyngeal cancer while protecting adjacent vital structures and reducing treatment toxicity (19–23). Moreover, due to dosimetric advantages (24), IMRT is also superior to 2DCRT in terms of preservation of parotid gland, improvement of quality of life (17, 21) and reduction of temporal lobe neuropathy (TLN) rate (20, 25) in patients with nasopharyngeal carcinoma. Patients with nasopharyngeal carcinoma treated with IMRT can achieve local control and overall survival rates of up to 90% and 80%, respectively (26, 27), which are better than those of 2DCRT (19, 22, 23, 28). **Table 1** summarized the clinical data on IMRT versus 2D-CRT (29).

**Table 1:** **The clinical data on IMRT ty 40versus 2D-CRT**.

Author	Year	Stage	Radiotherapy	No. (n)	Median age
Moon et al. (29)	2016	T1-4N0-3M0	IMRT	497	–
			2D-RT	350	–
Kam et al. (20)	2007	T1-2bN0-1M0	IMRT	28	45.5
			2D-RT	28	50.5
Lai et al. (30)	2011	M0	IMRT	512	–
			2D-RT	764	–
Peng et al. (19)	2012	M0	IMRT	306	46.7
			2D-RT	310	44.8
Qiu et al. (31)	2017	M0	IMRT	102	–
			2D-RT	74	–
Tang et al. (32)	2015	M0	IMRT	540	44.5
			2D-RT	512	44.5
Zhang et al. (33)	2015	M0	IMRT	2245	–
			2D-RT	4836	–
Zhou et al. (25)	2013	M0	IMRT	506	–
			2D-RT	747	–
Zhong et al. (34)	2013	T1-2bN0-2M0	IMRT	32	–
			2D-RT	37	–
Lee et al. (35)	2014	M0	IMRT	444	52
			2D-RT	434	48
Du et al. (22)	2019	M0	IMRT	5212	–
			2D-RT	8092	–
Author	RT dose of tumor (Gy)	Results (IMR vs 2D-CRT)			
		Clinical outcomes		Side effects	
Moon et al. (29)	69.49(± 3.18)	5-year OS: 76.7 % vs 59.7 % (p < 0.001); in T3–4 subgroup,5-year OS: 70.7% vs 50.4 % (p ≤ 0.001)		–	
	69.58 (±3.34)				
Kam et al. (20)	66 ± BT	–		delayed xerostomia:39.3% vs 82.1%, P =0.001; stimulated parotid flow:0.90 vs 0.05, P<0.0001; stimulated whole saliva flow:0.41 vs 0.20, P =0.001	
	66 ± BT				
Lai et al. (30)	60–64	5-year LRFS: 92.7% vs 86.8%; 5-year NRFS: 97.0% vs 95.5%; 5-year DMFS: 84.0% vs 82.6%; 5-year DFS: 75.9% vs 71.4%		–	
	68–76				
Peng et al. (19)	74 ± BT	5-year actuarial local control rate: 90.5% vs 84.7%; 5-year NRFS: 92.4% vs 92.9% (p > 0.05); 5-year OS: 79.6% vs 67.1%(p = 0.001); in T3 group, local control rate:91% vs 81.5%;in T4 group, local control rate: 80% vs 62.2%; in N2 group, NRFS:93.9% vs 91.4% (p = 0.02)		–	
	70–74 ± BT				
Qiu et al. (31)	62–70	5 year-OS: 90.4% vs 76.1% (P = 0.007); 5 year-DFS: 85.7% vs 71.2% (P = 0.029); 5 year-LRRFS: 97.9 vs 88.3% (P = 0.049)		Grade 2–4 xerostomia:34.3% vs 52.7(P = 0.015); hearing loss:22.5% vs 40.5(P = 0.010)	
	66–80				
Tang et al. (32)	68	IMRT improved LRFS and OS (P<0.001, P<0.001, respectively)		–	
	68–76				
Zhang et al. (33)	68	5 year-LRFS: 95.6% vs 90.8%; 5 year-LRRFS: 92.5% vs 88.5%; 5 year-PFS: 82.1% vs 76.7%; 5 year-OS: 87.4% vs 84.5% (P<0.001).5 year-DMFS: 87.6% vs 85.7% (P = 0.056); 5 year-NRFS: 96.3% vs 97.4% (P = 0.217).		–	
	68–76				
Zhou et al. (25)	68	–		5-year incidence of TLI: 16% vs 34.9% (P<0.001)	
	68–76				
Zhong et al. (34)	70	–		1-year incidence of dry mouth: 9.38% vs 94.59%(P<0.01); 1-year incidence of difficulty in opening mouth: 6.25% vs 72.97% (P < 0.01)	
	70				
Lee et al. (35)	70	5-year DSS: 85% vs 78%		neurological toxicity rate: 1.8% vs 7.4%	
	66				
Du et al. (22)	60-74	5-year OS: OR=1.70, 95%CI=1.36–2.12; 5-year LRFS: OR=2.08, 95%CI=1.82–2.37; 5-year PFS: OR=1.40, 95%CI=1.26-1.56		late xerostomia: OR =0.21, 95%CI=0.09–0.51; trismus: OR=0.16; 95%CI=0.04–0.60; TLN: OR=0.40, 95%CI=0.24–0.67	
	66-80				

OS: overall survival; LRFS: local relapse-free survival; NRFS: nodal relapse-free survival; DMFS: distant metastasis-free survival; DFS: disease-free survival; TLI: radiation-induced temporal lobe injury; LRRFS: loco-regional relapse-free survival; PFS: progression-free survival; DSS: disease-specific survival.

## 3 Interruption of radiotherapy

In order to achieve better prognosis, an uninterrupted routine radiotherapy schedule is an essential necessity for precise radiotherapy of nasopharyngeal carcinoma (36). Disruptions in radiotherapy, however, are inevitable for several unavoidable factors, such as public holidays (37) (the largest share, about 39-46%), machine failures, patient compliance, and adverse effects of treatment (38). Similarly, because of the pandemic of Corona Virus Disease 2019, confirmed positive patients had longer treatment interruptions, which led to fewer patients completing radiotherapy, thus increasing local disease progression (39).

The length of delayed treatment is a key indicator of the severity of treatment interruption (36, 40). It has been shown that 5-year survival is reduced by 10-20% in patients with squamous head and neck cancer who are treated for a total duration of up to 10 days beyond the original schedule, and even a one-day interruption results in 1.4% reduction in local control (41). The timing of radiotherapy interruption is of course important (42). Skladowsky et al. (43) reported that patients with supraglottic laryngeal carcinoma who interrupted radiotherapy on day 19 had lower local tumor control than those without a treatment gap. Generally speaking, nasopharyngeal cancer is extremely sensitive to radiotherapy. Interruption of radiotherapy or prolongation of treatment can have an adverse effect on the prognosis of patients (44, 45).

### 3.1 Split-course radiotherapy

Split-course radiotherapy is a form of radiotherapy fractionation. In contrast to conventional radiotherapy, the single dose is greater, the total radiotherapy dose is lower, and radiotherapy sessions is less frequent. Split-course radiotherapy is usually given at high doses of 3-5 Gy per day or even higher (46). Split-course radiotherapy is usually divided into two courses, typically 1-2.5 weeks, with an interval of 4-6 weeks between treatments, that can increase the total treatment time. Effectiveness and tolerability are assessed by the physician during this interval. Recovery of normal tissue also occurs during this interval, which reduces the incidence of acute grade ≥3 toxicity to 41-53% (47).

Some studies have shown that the poorer efficacy of split-course radiotherapy in comparison to continuous radiotherapy (48–50) may be related to the interruption of treatment during the interval and the accelerated repopulation of malignant cells, which leads to reduced efficacy (47, 51).

### 3.2 Length of interruption time

The study by Kong et al. found median interruption time of 3 days was detrimental for prognosis (3-year OS: 94.4% *vs* 64.2%, P=0.046) (52). In the study by Xu et al, patients with nasopharyngeal cancer were analyzed for the effects of interruptions >2 days *vs* ≤ 2 days, >3 days *vs* ≤ 3 days, and 4 days *vs* ≤ 4 days on LFRS, PFS, and OS, respectively. The results demonstrated that the interruption time threshold of 4 days had significant influence on PFS (1-year PFS:92.9% *vs* 91.2%; 3-year PFS:72.1% *vs* 81.9%; P=0.010), and OS (1-year OS:97.6% *vs* 97.4%; 3-year OS:80.8% *vs* 87.9%; P=0.002) (53). In the prognostic study of IMRT combined with or without chemotherapy in patients with nasopharyngeal cancer by Shyh-An Yeh et al (54), we could find that radiotherapy interruption (≥5 days) was a poor prognostic factor for overall survival (OS) (5-year OS: 83.4% *vs* 67.8%, P=0.007). Another study by Yao et al. also found interruption of radiotherapy for more than 5 days in nasopharyngeal cancer patients with stage T3-T4 was an unfavorable factor impacting prognosis (5-year LRFS: 97% *vs* 83%, P < 0.001; multivariate analysis: HR = 9.64, 95% CI= 4.10-22.65). Besides, patients receiving a schedule dose of 70 Gy in 33 fractions (2.12 Gy/F) were significantly (P = 0.013) more likely to have a longer radiotherapy interruption (> 5 days) than patients who received a dose of 68 Gy in 30 F (2.27 Gy/F) (36). While another study showed that interruption of more than 7 days was detrimental for prognosis (training cohort: 5-year OS: 82.4% *vs* 86.5%, P = 0.001; validation cohort: 5-year OS, 85.2% *vs* 86.7%, P = 0.013). Time of interruption was also confirmed as an independent prognostic factor by further multifactorial analysis (training cohort: HR= 1.49, 95% CI=1.14-1.95, P = 0.003; validation cohort: HR=1.37, 95% CI=1.07-1.65, P=0.031) (55).

### 3.3 Stages in which radiotherapy interruptions occur

Kwong et al. briefly explored the time point at which radiotherapy interruptions occurred throughout the course of treatment and found that interruptions occurring at or near the beginning of treatment did not significantly affect prognosis. Besides, they also found that the rate of loco-regional failure increased by 3.3% for each day of treatment interruption (56). In the study of Yang et al., patients were categorized into prior and subsequent interruptions based on whether they were halfway through their radiotherapy schedule, and were subsequently grouped again according to the duration of the interruption. The results showed that prior interruptions longer than 1 day (5-year OS: 89.6% *vs*. 85.7%, p<0.001; 5-year DFS: 81.4% *vs*. 76.4%, p<0.001) and subsequent interruptions longer than 4 days (88.4% *vs*. 82.3%, p<0.001; 79.2% *vs*. 75.1%, p=0.006) were significantly detrimental to DFS and OS. In the further multifactorial analysis, interruptions longer than 3 or 4 days afterwards were both poor prognostic factors (57). Certainly, it has been reported that the prolongation of the treatment time has no effect on the prognosis of the patients (57).

The time of treatment initiation is also critical, with the exception of factors such as prolonged radiotherapy and interruption of radiotherapy that can negatively affect patient outcomes. One study showed that for each additional week of time between diagnosis and formal initiation of treatment for head and neck cancer patients, their local control rate decreased by 1%. And, after waiting 28 days (the median waiting time), 62% of patients had a 46% increase in tumor volume and 20% had metastases to lymph nodes (58, 59). Evan M et al. also comprehensively analyzed the effect of delayed treatment on the prognosis of patients with head and neck cancer and concluded that the delay in the time from diagnosis to treatment and the prolonged postoperative wait for adjuvant radiotherapy could adversely affect the prognosis of patients (60). A short postoperative interval to adjuvant radiotherapy was found to be beneficial in improving patient survival, and this interval was usually considered optimal to be controlled at 6 weeks or less (61–63). **Table 2** summarized real-world data on the impact of radiotherapy interruptions.

**Table 2:** Real-world data on the impact of radiotherapy interruptions.

Author	Year	No. (n)	Stage	Cutoff
Xu et al. (45)	2010	1706	I-IV	RT interruption vs non-interruption
Wang et al. (44)	2014	695	I-IVA	–
Kong et al. (52)	2018	32	III-IVB	–
Xu et al. (53)	2017	515	I-III	≤4 vs >4 days
Yeh et al. (48)	2021	326	I-IVA	≤5 vs >5 days
Yao et al. (49)	2019	7826	–	<7 vs ≥7 days
Yang et al. (51)	2021	4510	I-IVA	preceding interruptions <1 vs ≥1 days or latter interruptions <4 vs ≥4 days
Author	Results			
	Clinical outcomes			Multivariate analysis (RT interruption)
Xu et al. (39)	5-year OS: 51.7% vs 69.5%, P<0.0001			unfavorable factor
Wang et al. (38)	–			LRC: HR=5.481, P<0.001; OS: HR=4.233, P<0.001
Kong et al. (46)	3-year OS: 64.2%vs 94.4%, P=0.046			–
Xu et al. (47)	3-year PFS: 81.9% vs 72.1%, P<0.05; 3-year OS: 87.9% vs 80.8%, P<0.05			LRFS: HR=1.047(0.512-2.142), P=0.900; PFS: HR=1.488(1.012-2.188), P=0.043; OS: HR=1.741(1.135-2.668), P=0.011
Yeh et al. (48)	5-year OS: 83.4% vs 67.8%, P<0.001; 5-year DFS: 75.3% vs 61.7%, P=0.001; 5-year LC: 92.8% vs 88.2%, P=0.164; 5-year DFF: 88.7% vs 78.5%, P=0.008			–
Yao et al. (49)	5-year OS: 86.5% vs 82.4%, P= 0.001(training cohort); 86.7% vs 85.2%, P = 0.013(validation cohort)			OS: HR=1.49, 95%CI=1.14-1.95, P=0.003(training cohort); HR=1.37, 95%CI=1.07-1.65, P=0.031(validation cohort)
Yang et al. (51)	preceding interruptions ≥1 days (5-year OS: 89.6% vs 85.7%, P<0.001; 5-year DFS:81.4% vs 76.4%, P<0.001); latter interruptions ≥4 days (5-year OS: 88.4% vs 82.3%, P<0.001; 5-year DFS: 79.2% vs 75.1%, P=0.006)			OS: HR=1.404; 95%CI=1.143-1.723, P=0.001; DFS: HR=1.351, 95%CI=1.105-1.652, P=0.003(latter interruptions ≥4 days)

## 4 Possible mechanisms

Tumor tissue regenerates at a faster rate than normal tissue, and the onset of rapid cell regeneration in tumor tissue during the treatment interval results in a lower radiobiologic dose to the planned target volume (PTV) (64). Radiotherapy interruption for nearly a full workweek and reduction in radiation service utilization may compromise the therapeutic benefit for patients because of the reduction in biological activity, which can lead to sublethal loss of repair (65).

**Figure 1:**
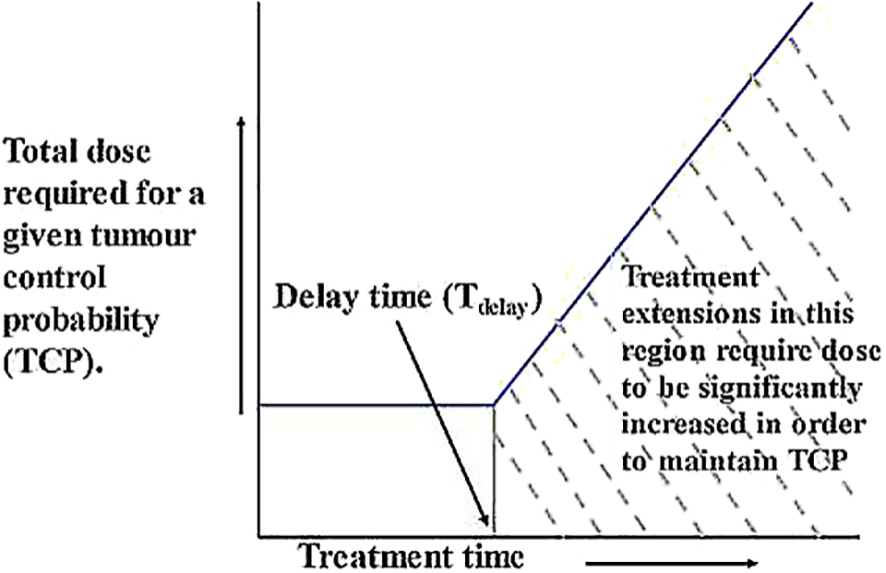
Graphical representation of the relationship between TCP and treatment duration. After the start of treatment, tumor repopulation remains close to zero, meaning that the dose required to maintain TCP remains essentially constant (horizontal line). After a delay of several weeks, the remaining cells begin to repopulate rapidly, and the additional dose required to kill new cells and maintain TCP increases linearly with time (67).

At the beginning of radiotherapy, numerous tumor cells will be far from the capillaries and will therefore be in various states of oxygen deprivation. They will either be in a quiescent state or multiply at a much slower rate than when they were initially created. In addition, cell loss factor (CLF) is usually high while treatment is starting, especially in larger tumors. Tumors become smaller as radiotherapy proceeds, vascular distribution begins to improve, and CLF decreases. As a result, any cells that have not been killed by radiation begin to become better oxidized and begin to grow (repopulate) at or near their fastest rate (66). This involves the well-known 4R principles of radiotherapy, namely regeneration, repair, reoxygenation and redistribution. The kinetics of tumor regeneration are graphically summarized in **Figure 1**, where the characteristic “dog-leg” shape shows that tumor repopulation remains close to zero after the start of treatment, meaning that the dose required to maintain TCP is essentially constant (horizontal line). After a delay of several weeks, the remaining cells begin to repopulate rapidly, and the additional dose required to kill new cells and maintain TCP increases linearly with time. Therefore, the uncompensated interruptions that lead to the extension of treatment to this period are particularly problematic (67). Unless additional doses are added, eradication of newly generated cells becomes unlikely and tumor progression is thus possible.

The time to tumor multiplication (Tpot) is an important issue in cancer treatment. A study by Delahaut et al. revealed a mean absolute tumor progression rate of 0.23 ± 0.2 cm^3^/day in 19 patients with squamous cell carcinoma of the head and neck (68). In more aggressive tumors, Tpot values may be surprisingly low, usually much less than 7 days, and re-proliferation rates are much faster (69–71). The delay of starting radiotherapy may lead to residual tumor proliferation after surgery. Besides, any tumor cells which were still existing at the end of the treatment are likely to grow at the fastest rate. If treatment is prolonged at this point, the increased time will allow for the generation of more cells.

Unless additional doses are added, eradication of newly generated cells becomes unlikely and tumor progression is thus possible. The kinetics of tumor regeneration are graphically summarized in **Figure 1**, where the characteristic “dog-leg” shape shows that tumor repopulation remains close to zero after the start of treatment, meaning that the dose required to maintain TCP is essentially constant (horizontal line). After a delay of several weeks, the remaining cells begin to repopulate rapidly, and the additional dose required to kill new cells and maintain TCP increases linearly with time. Therefore, the uncompensated interruptions that lead to the extension of treatment to this period are particularly problematic (67).

## 5 Measures that can be taken

### 5.1 Preventive measures

Nutritional assessment prior to treatment is also quite important. Some studies have shown that patients with pre-treatment malnutrition were significantly more likely to interrupt treatment than patients with normal nutrition (72). Oral prophylactic nutritional supplements can improve patient tolerance to concurrent radiotherapy (73). Oral care issues should not be underestimated either (74). Oral mucositis is a common toxic side effect during concurrent radiotherapy for nasopharyngeal cancers.

Adequate protein intake minimizes the severity of oral mucositis in patients with head and neck cancer undergoing radiotherapy (75). Early nutritional intervention, including oral feeding, nasogastric tube, gastrostomy, etc., can significantly improve weight loss and interrupt or delay of radiotherapy (76). Parenteral supplementation with glutamine (bipeptide) can also significantly reduce the rate of treatment interruption and the incidence of serious adverse reactions (77). The standard treatment of malnutrition should follow the five-step treatment principle of the European Society for Clinical Nutrition and Metabolism (ESPEN). ONS is the preferred, but not the only, form of enteral nutrition recognized by guidelines and expert consensus. It is also quite essential to establish good relationship with patients and pay attention to their psychological problems. According to Chen et al, being depressed before treatment was significantly associated with radiotherapy interruption and low survival in head and neck cancer patients (78). Pre-treatment should also focus on the mental health and mental status of patients. Patients with depressive symptoms are also more likely to have disrupted treatment, so it is important to focus on screening for depression and timely intervention during treatment (79).

### 5.2 Compensation for radiotherapy interruption

Treatment interruptions are inevitable, especially in the context of a new coronary pneumonia pandemic. For how to compensate for interrupted doses, Hendry et al. made the following recommendations: a. Use weekend time for radiotherapy; b. Increase the number of daily radiotherapy sessions, such as splitting twice a day; c. Increase the dose of a single radiotherapy session without extending the total treatment time; and d. Extend the total treatment time (80). However, regimen b increases the probability of normal tissue complications due to incomplete repair of normal tissue between divisions, resulting in increased sublethal damage to normal tissue (64). Regimens c and d either result in reduced local control rates or excessive late adverse effects (80).

The risk of radiotherapy interruption should be taken into account when the treatment regimen is developed and a set of compensatory measures, such as an increased compensatory dose (65), i.e., equivalent dose of 2 Gy per fraction (EQD2) (81), should be developed based on the physical condition of the patient, the severity of the disease. EQD2VH can be used as a decision tool when making a decision on the most appropriate compensation package for patients. It provides radiobiological dose-volume histograms that explain inhomogeneous dose distributions, as well as direct visual and quantitative comparisons between the plan being studied and the expected plan. Key dose-volume histogram statistics are provided for each plan to help monitor dose and compare with dose limits (64).

Dose compensation usually takes into account the histologically relevant factors κ (Gy/d) (41) and trigger time TK (67) for accelerated cell repopulation of 28 days. Monte Carlo simulations, which quantifies the biological effects of radiotherapy interruptions as well as assessing statistical uncertainty, are available to provide time factor κ (Gy/d) algorithm, assess the daily rate of BED decline, and calculate the residual fractionated dose to guide the subsequent treatment (41, 80, 82). It has been demonstrated that prolonged total treatment time is associated with decreased local control rates in head and neck cancers (83). The same can be inferred for other tumors, particularly in cases with high tumor growth rates (41, 80, 82).

Systemic therapy is also an appropriate option (84). Reducing the negative impact of radiotherapy interruption relies not only on a flexible response from radiation oncologists, but also on appropriate comprehensive care and dedicated multidisciplinary collaboration (85).

## 6 Conclusion

Radiotherapy interruption can have varying degrees of impact on patient outcomes, and the possibility of such interruptions should be minimized in actual clinical practice. However, due to the existence of some irresistible factors, sometimes radiotherapy interruption cannot be avoided. When radiotherapy is interrupted, remedial measures should be taken as much as possible. For example, increase the number or dose of radiotherapy, or combine other treatment modalities to reduce the adverse effects caused by radiotherapy interruption.

## Author contributions

FZ drafted the article. DY revised it critically. XL did final approval of the version to be submitted. All authors contributed to the article and approved the submitted version.
